# Mortality surrogates in combined pulmonary fibrosis and emphysema

**DOI:** 10.1183/13993003.00127-2023

**Published:** 2024-04-04

**Authors:** An Zhao, Eyjolfur Gudmundsson, Nesrin Mogulkoc, Coline van Moorsel, Tamera J. Corte, Pardeep Vasudev, Chiara Romei, Robert Chapman, Tim J.M. Wallis, Emma Denneny, Tinne Goos, Recep Savas, Asia Ahmed, Christopher J. Brereton, Hendrik W. van Es, Helen Jo, Annalisa De Liperi, Mark Duncan, Katarina Pontoppidan, Laurens J. De Sadeleer, Frouke van Beek, Joseph Barnett, Gary Cross, Alex Procter, Marcel Veltkamp, Peter Hopkins, Yuben Moodley, Alessandro Taliani, Magali Taylor, Stijn Verleden, Laura Tavanti, Marie Vermant, Arjun Nair, Iain Stewart, Sam M. Janes, Alexandra L. Young, David Barber, Daniel C. Alexander, Joanna C. Porter, Athol U. Wells, Mark G. Jones, Wim A. Wuyts, Joseph Jacob

**Affiliations:** 1 Satsuma Lab, Centre for Medical Image Computing, UCL, London, UK; 2 Centre for Medical Image Computing, UCL, London, UK; 3 Department of Respiratory Medicine, Ege University Hospital, Izmir, Turkey; 4 Interstitial Lung Diseases Center of Excellence, Department of Pulmonology, St Antonius Hospital, Nieuwegein, Netherlands; 5 Department of Respiratory Medicine, Royal Prince Alfred Hospital and University of Sydney, Sydney, Australia; 6 Department of Radiology, Pisa University Hospital, Pisa, Italy; 7 Interstitial Lung Disease Service, Department of Respiratory Medicine, University College London Hospitals NHS Foundation Trust, London, UK; 8 NIHR Southampton Biomedical Research Centre and Clinical and Experimental Sciences, University of Southampton, Southampton, UK; 9 BREATHE, Department of Chronic Diseases and Metabolism, KU Leuven, Leuven, Belgium; 10 Department of Respiratory Diseases, University Hospitals Leuven, Leuven, Belgium; 11 Department of Radiology, Ege University Hospital, Izmir, Turkey; 12 Department of Radiology, University College London Hospitals NHS Foundation Trust, London, UK; 13 Institute of Lung Health and Immunity (LHI) / Comprehensive Pneumology Center (CPC), Helmholtz Zentrum München, Munich, Germany; 14 Department of Radiology, Royal Free London NHS Foundation Trust, London, UK; 15 Department of Radiology, Royal United Hospitals Bath NHS Foundation Trust, Bath, UK; 16 Division of Heart and Lungs, University Medical Center, Utrecht, Netherlands; 17 Queensland Centre for Pulmonary Transplantation and Vascular Disease, The Prince Charles Hospital, QLD, Australia; 18 School of Medicine & Pharmacology, University Western Australia, WA, Australia; 19 Fiona Stanley Hospital, Perth, Australia; 20 Antwerp Surgical Training, Anatomy and Research Centre (ASTARC), Faculty of Medicine and Health Sciences, University of Antwerp, Edegem, Belgium; 21 Cardiovascular and Thoracic Department, Pisa University Hospital, Pisa, Italy; 22 National Heart and Lung Institute, Imperial College London, London, UK; 23 Lungs for Living Research Centre, UCL, London, UK; 24 Department of Neuroimaging, Institute of Psychiatry, Psychology and Neuroscience, King’s College London, London, UK; 25 Centre for Artificial Intelligence, UCL, London, UK; 26 Department of Respiratory Medicine, Royal Brompton Hospital, London, UK; 27 Imperial College London, London, UK

**Keywords:** Combined pulmonary fibrosis and emphysema, mortality surrogates, idiopathic pulmonary fibrosis, computed tomography

## Abstract

**Background:**

Idiopathic pulmonary fibrosis (IPF) with co-existent emphysema, termed combined pulmonary fibrosis and emphysema (CPFE) may associate with reduced forced vital capacity (FVC) declines compared to non-CPFE IPF patients. We examined associations between mortality and functional measures of disease progression in two IPF cohorts.

**Methods:**

Visual emphysema presence (>0% emphysema) scored on computed tomography identified CPFE patients (CPFE:non-CPFE: derivation cohort=317:183; replication cohort=358:152), who were subgrouped using 10%, or 15% visual emphysema thresholds, and an unsupervised machine learning model considering emphysema and ILD extents. Baseline characteristics, 1-year relative FVC and diffusing capacity of the lung for carbon monoxide (DLco) decline (linear mixed-effects models), and their associations with mortality (multivariable Cox regression models) were compared across non-CPFE and CPFE subgroups.

**Results:**

In both IPF cohorts, CPFE patients with ≥10% emphysema had a greater smoking history and lower baseline DLco compared to CPFE patients with <10% emphysema. Using multivariable Cox regression analyses in patients with ≥10% emphysema, 1-year DLco decline showed stronger mortality associations than 1-year FVC decline. Results were maintained in patients suitable for therapeutic IPF trials and in subjects subgrouped by ≥15% emphysema and using unsupervised machine learning. Importantly, the unsupervised machine learning approach identified CPFE patients in whom FVC decline did not associate strongly with mortality. In non-CPFE IPF patients, 1-year FVC declines ≥5% and ≥10% showed strong mortality associations.

**Conclusion:**

When assessing disease progression in IPF, DLco decline should be considered in patients with ≥10% emphysema and a ≥5% 1-year relative FVC decline threshold considered in non-CPFE IPF patients.

## Introduction

Emphysema is a common pulmonary finding on computed tomography (CT) imaging of idiopathic pulmonary fibrosis (IPF) patients [1]. The term combined pulmonary fibrosis and emphysema (CPFE) describes a potential clinical endotype characterized by the coexistence of upper lobe-predominant emphysema, lower lobe-predominant fibrosis and relative preservation of forced vital capacity (FVC) in the context of a disproportionately reduced gas transfer (diffusing capacity of the lung for carbon monoxide, DLco) [1–3]. CPFE is highly heterogeneous in terms of the distribution and relative extents of fibrosis and emphysema seen on CT.

CPFE patients are typically categorised using visual thresholds of emphysema extent: >0%, ≥5%, ≥10%, ≥15%. It has been suggested that a subset of CPFE patients (≥15% emphysema) may manifest slower rates of FVC decline than CPFE patients with lesser amounts of emphysema [4]. Despite the importance of fibrosis in driving FVC decline, fibrosis extent hasn’t been considered in prior definitions of CPFE [5]. Categorisation of CPFE patients using a combination of fibrosis and emphysema is possible using data-driven machine learning methods. SuStaIn [6] is a machine learning method initially proposed for subtyping and modelling disease progression behaviour in dementia, which has been extended to COPD [7]. SuStaIn can identify disease subtypes with different progression patterns and can reconstruct their progression trajectories from cross-sectional data. A by-product of this approach would be the identification of patients in different CPFE subtypes who may benefit from different forms of disease progression monitoring, which in turn could inform clinical trial design.

In our study, we hypothesised that FVC decline, the most widely used surrogate for mortality prediction in IPF might show limited associations with mortality in independent CPFE populations with ≥10% and ≥15% emphysema scored visually on CT imaging, and in CPFE subgroups categorised by considering relative extents of interstitial lung disease (ILD) and emphysema. We further hypothesised that DLco decline could represent an alternative surrogate for mortality in IPF patients with CPFE [5, 8].

## Methods

### Cohorts

Two independent IPF cohorts diagnosed by multidisciplinary teams were studied. Patients with infection or cancer on baseline CT or who died within 3 months of the baseline CT were excluded from the study. We studied two IPF cohorts so as to test whether DLco could be a consistent mortality surrogate in independent IPF populations. The derivation cohort (n=500) derived from three centres: Ege University Hospital, Izmir, Turkey; St Antonius Hospital, Nieuwegein, Netherlands; Pisa University Hospital, Italy. The replication cohort (n=510) derived from four centres: University Hospital Southampton NHS Foundation Trust, UK; University College London Hospitals NHS Foundation Trust, UK; University Hospitals Leuven, Belgium; Australian IPF registry, Australia. CONSORT diagrams for derivation cohort and replication cohort are shown in Supplementary Figure 1. Approval for this retrospective study of clinically indicated pulmonary function and CT data was obtained from the local research ethics committees and Leeds East Research Ethics Committee: 20/YH/0120.

### Visual CT Scoring of Emphysema and ILD

Emphysema extent and fibrosis extent were visually scored in 6 lobes (the lingula was counted as the sixth lobe) by an experienced thoracic radiologist (JJ) with 16 year’s experience. Fibrosis extent comprised the sum of ground glass density (with overlying reticulation or traction bronchiectasis), reticulation, traction bronchiectasis and honeycomb cysts. Lobar extents of emphysema/fibrosis were summed and divided by 6 to obtain a lung percentage of emphysema/fibrosis.

**Figure 1:**
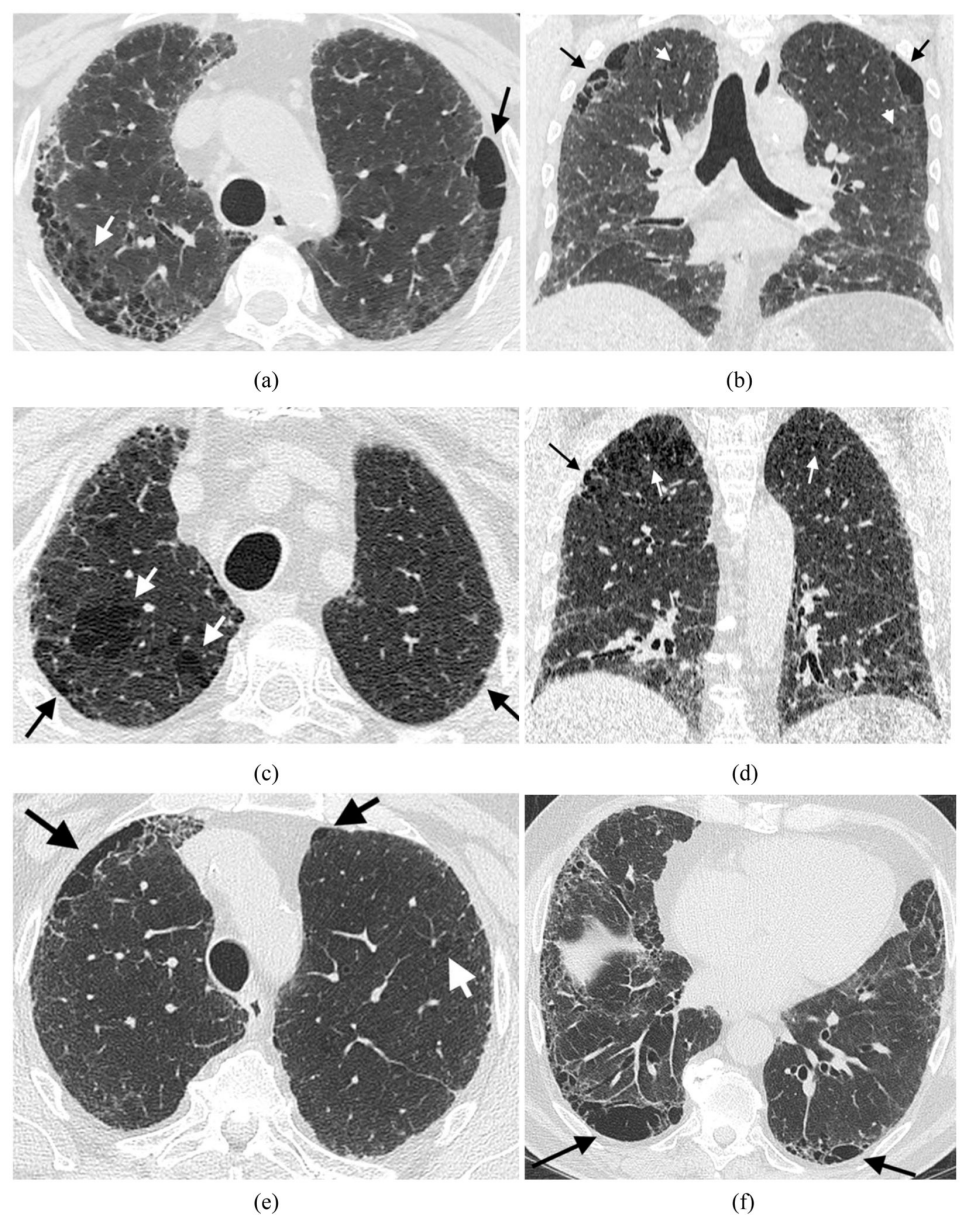
Computed tomography images of three subjects with 10% emphysema scored visually. A 59-year-old male 5-pack-year ex-smoker with axial (a) and coronal (b) imaging shows extensive upper lobe paraseptal emphysema (black arrows) and also centrilobular emphysema (white arrows) in a predominantly upper lobe distribution. Fibrosis with traction bronchiectasis, ground glass opacification and reticulation is seen in a lower zone predominant distribution. Figure c+d show respectively axial and coronal images of mixed paraseptal (black arrows) and centrilobular emphysema (white arrows) in a 60-year-old male 17-pack-year ex-smoker. Axial images in a 72-year-old male 20-pack-year ex-smoker demonstrate a predominantly paraseptal distribution of emphysema (black arrows) in the upper (e) and lower (f) lobes with minimal centrilobular emphysema (white arrow).

For the purposes of this study, a patient was defined as having CPFE is they had any emphysema on a CT. CPFE patients were subdivided in a primary analysis into those ≥10% emphysema (Figure 1), and in a secondary analysis into those ≥15% emphysema. CT imaging in a random subset of 122 subjects was evaluated independently by two radiologists (GC and JB: 3 and 4 years imaging experience respectively) to provide an estimate of observer variation for semi-quantitative scores of emphysema extent.

## Statistical analysis

Data are presented as means and standard deviations unless otherwise stated. Two-sample t-tests were used for continuous variables, and chi-squared tests were used for categorical variables. Kaplan-Meier survival plots and the log-rank test were used to test for differences in survival between non-CPFE IPF patients, and CPFE patients in different subgroups (using emphysema thresholds or SuStaIn subtype) in both IPF cohorts. Subanalyses were performed for patients satisfying lung function criterion for inclusion into IPF therapeutic trials (percent predicted DLco >30%, percent predicted FVC >50%, and forced expiratory volume in the first second/FVC ratio >0.7).

### FVC/DLco Decline Modelling

Linear mixed-effects (LME) models estimated absolute and relative 1-year FVC decline and 1-year DLco decline. The trajectory of FVC for patients from different countries/centres was modelled separately by using the LME model. Fixed effects included: age at baseline CT date, sex, smoking history (never vs. ever), antifibrotics (never vs. ever), baseline percent predicted FVC (nearest to and within 3 months of baseline CT date), and time since baseline CT imaging date. Each subject had a random intercept and random slope. FVC measurements between baseline FVC date and 18 months after baseline CT date were used to build the LME model. Subjects were required to have had an FVC measurement within 3 months of the CT, and at least one further follow up FVC measurement to be included in this analysis. Absolute and relative 1-year FVC declines were calculated. For relative 1-year FVC decline, each follow-up FVC measurement (mls) was divided by baseline FVC (mls) and multiplied by 100 [9] and LME-predicted relative FVC percentage calculated at 1 year. 1-year DLco decline was estimated using similar methods, with longitudinal DLco and baseline percent predicted DLco used in the LME models. LME models were implemented with MATLAB (version R2019b, Mathworks, Natick, Massachusetts, US).

### Machine Learning Delineation of CPFE Subtypes

Only patients with emphysema scored visually in any lobe were considered for SuStaIn CPFE analysis. Using baseline data alone, SuStaIn can identify disease subtypes with distinct progression trajectories that describe the evolution of multiple biomarkers. The progression trajectory for an individual disease subtype follows a linear z-score model, in which each biomarker is modelled as a monotonically increasing piece-wise linear function [6, 7]. Specifically, we used visually estimated fibrosis and emphysema extents within each of the six lobes as biomarkers (12 biomarkers in total). The extent of each biomarker was divided by the interobserver variability (calculated using the single determination standard deviation) of the biomarker as scored by two radiologists resulting in corresponding z-scores for the SuStaIn model. The z-score indicates an abnormal level of a biomarker and the piece-wise linear trajectory of each biomarker describes a continuous accumulation of abnormality: z-score = 0, 1, …, z_max_. z_max_ is the maximum z-score a biomarker can reach at the end stage of a disease and this maximum score can be a different number in different biomarkers. If we define the transition of a biomarker from one z-score to the next z-score as a z-score event, the trajectory of disease progression is a sequence of different z-score events in the various biomarkers under consideration.

The process of fitting of the SuStaIn model aims to find the optimal number of subtypes of disease, the proportion of each subtype within the population, and the order of z-score events for all biomarkers in each disease subtype. The trained SuStaIn model can then predict probabilities that an individual belongs to a particular subtype and stage [6].

An underlying assumption of SuStaIn is that the biomarkers will show a monotonic increase. As emphysema develops slowly, and IPF patients have a short survival time, it is less likely that an IPF patient without emphysema will develop emphysema during their lifetime. Accordingly, to avoid breaking the assumption that a biomarker will show a monotonic increase, only patients with emphysema scored visually in any lobe were considered for SuStaIn CPFE analysis.

### Cox Regression Modelling

In multivariable mixed-effects Cox regression models associations of FVC decline and DLco decline with mortality were examined across IPF subtypes. Models were adjusted for age, sex, smoking history (never vs. ever), antifibrotic use (never vs. ever), and baseline disease severity (using percent predicted DLco at baseline). Differences between different countries/centres in each cohort were modelled by assigning a random intercept for each centre. Cox models were used with a minimum of 8 outcome events per predictor covariate [10]. Cox regression models were tested for proportional hazards assumption using the Schoenfeld residuals test. The Concordance index (C-index) compared the goodness of fit of Cox regression models. P-values <0.01 were considered statistically significant. All mixed-effects Cox regression analyses were implemented by R (version 4.0.3 with Rstudio version 1.3.1093, Rstudio, Boston, Massachusetts, US).

### Group Comparisons for FVC and DLco Decline

To investigate the impact of emphysema on FVC and DLco decline in the different IPF subgroups (non-CPFE patients; CPFE patients classified using emphysema thresholds or SuStaIn), proportions of patients with ≥5% and ≥10% relative FVC decline in 1-year and ≥10% and ≥15% relative DLco decline in 1-year were calculated. Mean absolute 1-year FVC decline (mls) and DLco decline (mls/min/mmHg) were also calculated for the three subgroups. Analyses were performed in both IPF cohorts, with subanalyses in subjects fulfilling criteria for inclusion into IPF therapeutic trials. Chi-squared tests with Bonferroni-adjusted p-values were calculated for categorical variables. A one-way ANOVA test examined differences in mean absolute FVC decline (mls) with a post hoc Tukey Honest Significant Difference (HSD) test used to compare pairwise differences in subtypes.

## Results

### Baseline Characteristics

317/500 (63%) IPF patients in the derivation cohort had emphysema and were defined as CPFE compared to 358/510 (7%) IPF patients with CPFE in the replication cohort. CPFE patients were more likely to be smokers, had a higher percent-predicted FVC and lower percent-predicted DLco than non-CPFE patients.

Across the derivation and replication cohorts, CPFE patients with ≥10% emphysema comprised greater numbers of smokers and had lower baseline percent predicted DLco compared to CPFE patients with <10% emphysema (Table 1). To power analyses, patients in both IPF cohorts fulfilling entry criteria for therapeutic trials were combined into a single cohort (Supplementary Table 2). Baseline characteristics of CPFE patients with emphysema above or below 15% in derivation and replication cohorts are shown in Supplementary Table 3-4.

The interobserver variation in visual emphysema scores for the subset of 122 cases scored by two radiologists, measured using Cohens Kappa for 0%, 5%, 10%, and 15% emphysema thresholds was: 0.2, 0.5, 0.61, 0.69, respectively demonstrating substantial agreement for a 10% visual emphysema threshold.

### Machine Learning Model

**Figure 2:**
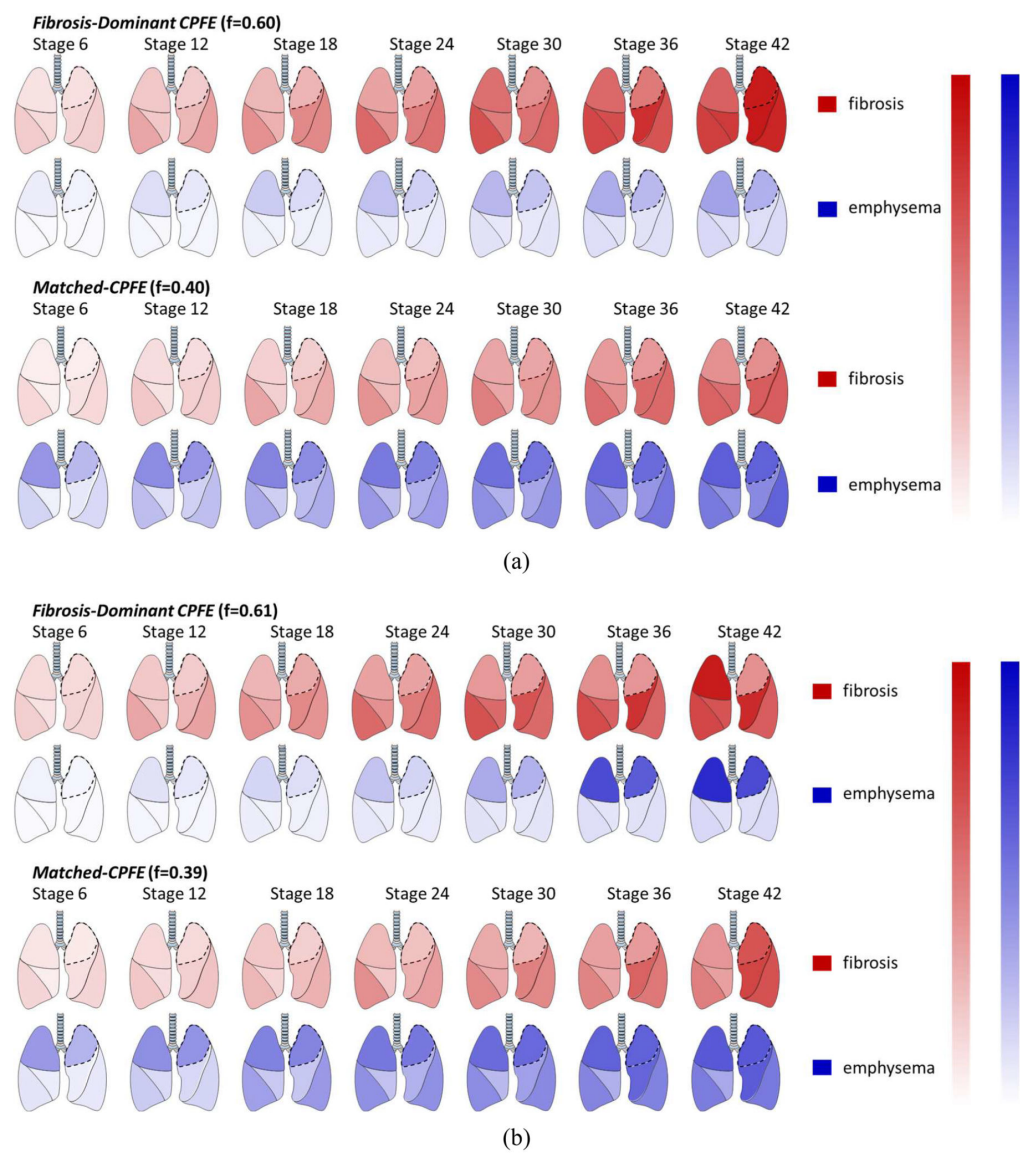
Identification of CPFE subtypes and subtype disease progression modelled by SuStaIn in the derivation cohort (a) and replication cohort (b). The rows show progression patterns of fibrosis extent (in red) and emphysema extent (in blue) in 6 lung zones (upper, middle and lower) in the two CPFE subtypes identified by SuStaIn: *Fibrosis-Dominant CPFE* and *Matched-CPFE*. Seven disease stages are highlighted, expressed as z-score intervals. In the *Fibrosis-Dominant CPFE* subtype comprising 60% of the derivation cohort and 60% of the replication cohort (top two rows in (a) and (b)), fibrosis is more severe at an early stage followed by a later emergence of emphysema. In the *Matched-CPFE* subtype comprising 40% of the derivation cohort and 39% of the replication cohort (bottom two rows in (a) and (b)), fibrosis and emphysema get worse together, with later stages showing relatively more extensive emphysema and less fibrosis compared to the *Fibrosis-Dominant CPFE* subtype. The upper lobe predominance of emphysema seen at early disease stages no longer exists in the later stages of the *Matched-CPFE* subtype. CPFE: combined pulmonary fibrosis and emphysema. This figure was produced with the assistance of Servier Medical Art (https://smart.servier.com).

Machine learning analyses of ILD and emphysema extents in the CPFE population identified two distinct CPFE subtypes. One subtype (*Fibrosis-Dominant CPFE*; 60% of derivation cohort CPFE patients and 61% of replication cohort CPFE patients) had much more extensive fibrosis at an early stage followed by a later emergence of emphysema (Figure 2). The second subtype (*Matched-CPFE*) demonstrated fibrosis and emphysema worsening together, with later stages showing relatively more extensive emphysema and less fibrosis compared to the *Fibrosis-Dominant CPFE* subtype (Supplementary Table 5 and 6).

### PFT Decline Analyses

Fewer CPFE patients with ≥10% emphysema reached the ≥10% or ≥5% 1-year FVC decline thresholds and had lower mean absolute FVC declines, though differences between groups did not reach statistical significance (Table 2). Greater numbers of CPFE patients with ≥10% emphysema demonstrated 1-year DLco declines ≥15%, though again results did not reach statistical significance (Table 3). Similar trends were found in the replication cohort, patients fulfilling criteria to enter IPF therapeutic trials (Table 2 and 3), and when CPFE was categorized using a 15% emphysema threshold or machine learning analyses (Supplementary Table 7 and 8).

### Survival Analyses

**Figure 3:**
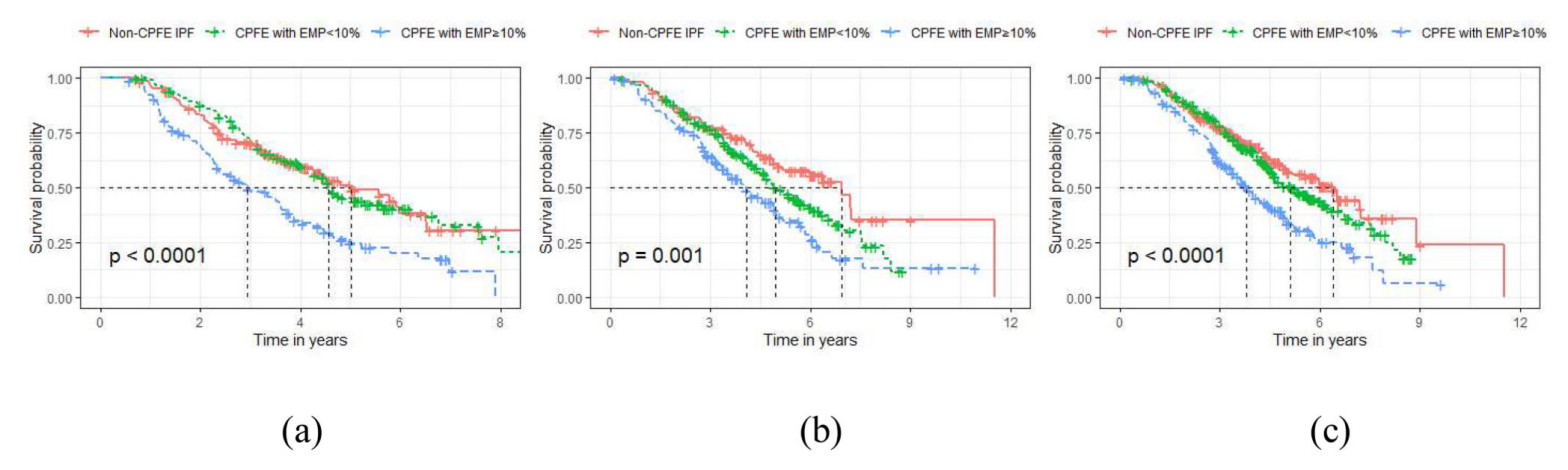
Kaplan-Meier curves of non-CPFE IPF patients (red), CPFE patients with emphysema <10% (green) and CPFE patients with emphysema ≥10% (blue) in the derivation cohort (a), the replication cohort (b), combined derivation and replication cohort patients qualifying for therapeutic trials (c). Log-rank tests show a significant difference in mortality between the three subtypes in all three analyses.

Kaplan-Meier survival plots (Figure 3) demonstrated that in both cohorts, non-CPFE and CPFE patients with <10% emphysema had a significantly better prognosis than CPFE patients with ≥10% emphysema. Results were maintained in patients fulfilling criteria to enter IPF therapeutic trials and were similar when CPFE patients were separated using a 15% emphysema threshold or machine learning analyses (Supplementary Figure 2 and 3).

### Mortality Analysis for Visual Emphysema Thresholds

Multivariable Cox regression models adjusted for patient age, sex, smoking history (never vs. ever), antifibrotic use (never vs. ever), and baseline percent predicted DLco showed that in non-CPFE patients, 5% and 10% 1-year FVC decline thresholds showed strong associations with mortality in derivation (5% 1-year FVC decline: HR=3.82, 95% CI=2.10-6.95, p<0.0001; 10% 1-year FVC decline: HR=4.26, 95% CI=2.42-7.50, p<0.0001) and replication (5% 1-year FVC decline: HR=2.72, 95% CI=1.43-5.19, p=0.002; 10% 1-year FVC decline: HR=2.73, 95% CI=1.37-5.44, p=0.004) cohorts (Table 4 and 5). Associations with mortality were maintained in patients fulfilling criteria to enter IPF therapeutic trials (5% 1-year FVC decline: HR=3.27, 95% CI=2.03-5.25, p<0.0001; 10% 1-year FVC decline: HR=4.36, 95% CI=2.69-7.06, p<0.0001; Supplementary Table 9).

For CPFE patients with ≥10% emphysema (derivation cohort n=103/352 (29%); replication cohort n=115/382 (30%)), in multivariable analyses, 1-year relative DLco decline showed a stronger association with mortality than 1-year relative FVC decline in derivation (DLco decline: HR=1.03, 95% CI=1.02-1.05, p<0.0001; FVC decline: HR=1.03, 95% CI=1.01-1.06, p=0.008) and replication (DLco decline: HR=1.03, 95% CI=1.01-1.05, p=0.001; FVC decline: HR=1.02, 95% CI=0.99-1.06, p=0.13) cohorts (Table 4 and 5). When DLco thresholds were examined in CPFE patients with ≥10% emphysema, ≥15% 1-year relative DLco decline showed stronger associations with mortality than ≥10% 1-year relative FVC decline in derivation (≥15% 1-year DLco decline: HR=2.67, 95% CI=1.64-4.35, p<0.0001; ≥10% 1-year FVC decline: HR=2.54, 95% CI=1.42-4.54, p=0.002) and replication (≥15% 1-year DLco decline: HR=3.88, 95% CI=2.12-7.10, p<0.0001; ≥10% 1-year FVC decline: HR=2.03, 95% CI=1.05-3.91, p=0.04) cohorts. In subjects eligible for inclusion into IPF therapeutic trials (where 144/589 (24%) patients had ≥10% emphysema) 1-year relative DLco decline (HR=1.04, 95% CI=1.03-1.06, p<0.0001) showed stronger associations with mortality than 1-year relative FVC decline (HR=1.05, 95% CI=1.02-1.08, p=0.0006) on multivariable Cox regression analyses (Supplementary Table 9). Similar trends were observed in multivariable analyses performed in CPFE patients with ≥15% emphysema (Supplementary Table 10-12).

### Mortality Analyses of Machine Learning Derived CPFE Subgroups

Trends seen for the 10% visual emphysema threshold were again replicated when CPFE patients were separated using machine learning analyses that considered ILD and emphysema extents. The *Matched-CPFE* cohort also delineated patients in whom FVC decline proved a poor surrogate for mortality. Importantly, in the *Matched-CPFE* cohort, DLco decline, whether measured as relative decline in percent-predicted DLco (derivation: HR=1.04, 95% CI=1.02-1.05, p<0.0001; replication: HR=1.03, 95% CI=1.01-1.05, p=0.001, clinical trial cohort: HR=1.04, 95% CI=1.03-1.06, p<0.0001) or a ≥15% DLco threshold (derivation: HR=2.63, 95% CI=1.54-4.52, p=0.0004; replication: HR=4.86, 95% CI=2.39-9.90, p<0.0001, clinical trial cohort: HR=3.61, 95% CI=2.16-6.02, p<0.0001) remained a strong surrogate for mortality (Supplementary Table 13-15). This was less evident for FVC decline (measured in mls) whether expressed as a continuous relative decline percentage (derivation: HR=1.04, 95% CI=1.01-1.07, p=0.006; replication: HR=1.02, 95% CI=0.99-1.06, p=0.23, clinical trial cohort: HR=1.06, 95% CI=1.03-1.09, p=0.0006) or a ≥10% FVC decline threshold (derivation: HR=2.48, 95% CI=1.22-5.07, p=0.01; replication: HR=2.36, 95% CI=1.14-4.91, p=0.02, clinical trial cohort: HR=2.67, 95% CI=1.42-5.02, p=0.002).

## Discussion

Our study evaluated functional indicators of disease progression in IPF patients with emphysema that have been the key mortality surrogates used in clinical care and therapeutic trials. We identified three important findings across two IPF populations: Firstly, we demonstrated the limited associations between relative FVC decline and mortality in CPFE patients with ≥10% and ≥15% emphysema, and conversely the strong associations with mortality for relative DLco decline in the same subgroups. Second, our machine learning model identified a subgroup of CPFE patients where a relatively greater amount of emphysema compared to ILD accentuated the limited associations between ILD-driven FVC decline and mortality in these CPFE patients. Lastly, in non-CPFE patients we showed that FVC decline is a powerful measure of IPF progression showing strong associations with mortality at both ≥5% and ≥10% 1-year FVC decline thresholds.

FVC decline occupies a cardinal role in the assessment of disease progression in IPF as it has been shown to be a strong surrogate for mortality [11]. The demonstration however that FVC decline may be curtailed in IPF patients with ≥15% [4] emphysema raised the question of whether FVC decline remained a surrogate for mortality in IPF patients with more extensive emphysema. Only one other study, by Schmidt et al [8], which was relatively underpowered (n=42) for subjects with moderate/severe emphysema (defined as emphysema at least as extensive as ILD), addressed this question and found that FVC decline did not associate with mortality at 12 months. Other studies considering IPF patients regardless of emphysema presence/extent have shown strong associations between mortality and other functional decline measures/thresholds including: DLco decline thresholds of ≥10% [12] and 15% [13], and FVC declines of ≥5% [14–16].

An explanation for the poor association between FVC decline and mortality in patients with more extensive emphysema may relate to the impact of fibrosis when encroaching on areas of emphysema. Emphysematous regions of lung commonly demonstrate air trapping as thickened small airways collapse on expiration. Fibrotic processes however can irreversibly pull open small airways. The supervening traction bronchiolectasis can result in emphysematous airspaces being ventilated, thereby artificially preserving FVC. In IPF patients with emphysema, as fibrosis progresses and extends to involve the upper zones of the lungs, more emphysematous lung may become incorporated into the expiratory lung volume over time. A consequence may be greater heterogeneity in expiratory lung volumes, superimposing considerable noise to an overarching pattern of progressive FVC decline. This effect is likely to be more pronounced in patients with more extensive emphysema.

One limitation in prior definitions of CPFE has been the focus on emphysema extent alone as the sole arbiter for categorising a CPFE endotype. A recent ATS/ERS/ALAT/JRS research statement identified a 5% emphysema threshold as a research definition for CPFE patients, whilst suggesting a 15% emphysema threshold for classifying a CPFE clinical syndrome [5]. In our study we found that a 10% emphysema threshold (which showed substantial CT observer agreement) may represent a better cut-off than a 15% emphysema threshold to identify a CPFE population disenfranchised by the use of FVC as a sole measure of disease progression.

A further challenge with CPFE definitions being determined by emphysema thresholds is that FVC decline is primarily driven by ILD progression rather than emphysema progression. Our unsupervised machine learning model (SuStaIn) considered both fibrosis and emphysema when subtyping patients and replicated the strong association of DLco decline and mortality in patients with more extensive emphysema seen in CPFE patients with ≥10% emphysema. By considering ILD extent in relation to emphysema extent, the SuStaIn model delineated of a subgroup of CPFE patients, fulfilling criteria to enter IPF therapeutic trials, where FVC decline did not associate strongly with mortality.

Prior studies have shown associations between DLco decline and mortality in IPF [8, 12, 13, 17–19] but have not analysed the impact of emphysema on DLco trends. DLco decline has generally been less consistent in its links with mortality than FVC decline in IPF patients [20]. Yet DLco decline may have particular relevance in subsets of IPF patients [21]. For example, the strong mortality signal for DLco decline seen in CPFE patients with more extensive emphysema could reflect progressive localised pulmonary hypertension complicating CPFE patients with more extensive emphysema [22, 23]. Our study findings suggest that in IPF patients with extensive emphysema a composite endpoint of FVC decline ≥10% or DLco decline ≥15% should be considered when assessing disease progression.

There were limitations to the current study. A single observer scored the CTs for fibrosis and emphysema. For studies to be clinically meaningful, they have to be suitably powered, and this requires the careful evaluation of large IPF populations. This is challenging with a current limited availability of radiologists and would occur more commonly in specialist ILD centres. The single read of CTs in this study aligns with other large scale IPF studies where pragmatic considerations required assessment of CTs by a single specialist [24, 25]. Similar functional measures and IPF subgroups proportions across both study cohorts provide reassurance for the validity of the visual CT scores. The improvement in observer agreement at higher emphysema thresholds (even amongst less experienced radiologists) adds confidence to the reliability of visual scores at an emphysema threshold of 1%. This also aligns with prior work [26] demonstrating improved interobserver agreement at emphysema extent categories of 10% and 15% versus 0% and 5%. The computer algorithm SuStaIn is not routinely available to clinicians at present, but was used to show the impact of considering ILD extent in the classification of CPFE subtypes. There was also missing data for longitudinal PFTs, reducing the sample size of both cohorts in the analyses of lung function decline. No imputation was performed however as we wanted the analyses to reflect the recorded functional status of the patients. Lastly, whilst we would have liked to have fully automated our machine learning model, using computationally quantified emphysema as an objective measure of disease, no existing automated tools can reliably distinguish emphysema from honeycombing and traction bronchiectasis.

In conclusion, annual relative DLco decline was shown to be a better mortality surrogate for patients with more than 10% emphysema than relative FVC decline. Findings were validated by a data-driven machine learning method that considers emphysema and ILD extents when defining patients with more extensive emphysema. These observations may be useful in clinical trial design to identify subjects where FVC decline is a poor disease progression measure. A 5% 1-year relative FVC decline threshold however was found to be a strong mortality indicator in non-CPFE IPF patients.

## Supplementary Material

Supplementary Materials

## Figures and Tables

**Table 1:** Baseline characteristics of non-CPFE IPF patients and CPFE patients with emphysema below or above 10% in the derivation and replication cohorts.

Cohort	Variable	Non-CPFE IPF patients	CPFE patients with emphy sema <10%	CPFE patients with emphysema >10%
Derivation cohort	Subjects (%)	183 (36.6)	174 (34.8)	143 (28.6)
	Age (years)	67.8±9.2	66.9±9.1	65.0±9.1
	Male (%)	110/183 (60.1)	143/174 (82.2)	132/143 (92.3)
	Never-/ever-smokers (ever %)	92/91 (49.7)	38/133 (77.8) *	8/134 (94.4) **
	Visual fibrosis extent (%)	38.7±14.6	36.3±14.1	40.8±13.5
	Visual emphysema extent (%)	0±0	4.8±2.3	20.4±8.8
	FVC (% predicted, n)	77.1±20.8 (158)	80.11±20.2 (150)	79.1±21.9 (122)
	DLco (% predicted, n)	52.2±16.5 (151)	51.6±15.1 (138)	40.4±13.33 (116)
Replication cohort	Subjects (%)	152 (29.8)	206 (40.4)	152 (29.8)
	Age (years)	71.6±8.4	71.9±8.3	70.5±8.0
	Male (%)	96/152 (63.2)	168/206 (81.6)	128/152 (84.2)
	Never-/ever-smokers (ever %)	78/74 (48.7)	51/152 (74.9) ^†^	22/129 (85.4) ^††^
	Visual fibrosis extent (%)	34.0±14.9	34.6±12.8	37.8±12.4
	Visual emphysema extent (%)	0±0	4.9±2.4	21.1±11.1
	FVC (% predicted, n)	84.5±21.1 (137)	84.4±20.5 (184)	86.6±18.9 (137)
	DLco (% predicted, n)	55.2±15.1 (121)	51.2±16.0 (176)	40.7±11.2 (126)

FVC: forced vital capacity; DLco: diffusing capacity of the lung for carbon monoxide; CPFE: combined pulmonary fibrosis and emphysema; IPF: idiopathic pulmonary fibrosis; * 171 patients and ** 142 patients had smoking data available in derivation cohort;

* 171 patients and ** 142 patients had smoking data available in derivation cohort;

^†^ 203 patients and ^††^ 151 patients had smoking data available in replication cohort.

**Table 2:** FVC decline analysis in different subgroups of IPF patients.

Cohort	Subgroup	FVC data available cases/all case	Relative 1-year FVC decline (%)		Absolute 1-year FVC decline (mls)	
			Number of ≥10% (proportion)	Number of ≥5% (proportion)	Mean	95% CI of difference between subgroups
Derivation cohort	Non-CPFE	150/183	51 (34%)	81 (54%)	163.50	-117.78~84.55*
	CPFE with emphysema <10%	136/174	39 (28.68%)	69 (50.74%)	180.12	-39.83~171.96^#^
	CPFE with emphysema ≥10%	115/143	27 (23.48%)	49 (42.61%)	97.43	-190.92~25.55^^^
Replication cohort	Non-CPFE	124/152	24 (19.35%)	50 (40.32%)	110.65	-85.47~41.54*
	CPFE with emphysema <10%	170/206	37 (21.76%)	75 (44.12%)	132.62	-44.55~90.45^#^
	CPFE with emphysema ≥10%	130/152	21 (16.15%)	44 (33.85%)	87.71	-107.57~17.74^^^
Combined drug trial cohort	Non-CPFE	222/236	59 (26.58%)	105 (47.30%)	142.94	-86.52~42.79*
	CPFE with emphysema <10%	240/261	57 (23.75%)	113 (47.08%)	164.81	-42.64~104.13^#^
	CPFE with emphysema ≥10%	150/157	29 (19.33%)	56 (37.33%)	112.19	-124.88~19.65^^^

The proportions of patients with more than 10% and 5% relative 1-year FVC decline, and the mean of absolute 1-year FVC decline in derivation, replication cohorts and combined drug trial cohort (patients fulfilling criteria to enter IPF therapeutic trials in derivation and replication cohorts) are shown in this table. The number of subjects with available FVC decline versus the number of all subjects belonging to a certain subgroup is shown in n/n format. We also compared a) non-CPFE with CPFE with emphysema <1%, b) non-CPFE with CPFE with emphysema ≥1%, c) CPFE with emphysema ≥10% and CPFE with emphysema <1%, in terms of the relative decline and absolute decline. We use *, # and ^ to denote comparison a), b), c) respectively in the table. **None of the comparisons showed statistically significant differences**. CPFE: combined pulmonary fibrosis and emphysema; IPF: idiopathic pulmonary fibrosis; FVC: forced vital capacity; CI: confidence interval.

**Table 3:** DLco decline analysis in different subgroups of IPF patients.

Cohort	Subgroup	DLco data available cases/all case	Relative 1-year DLco decline (%)		Absolute 1-year DLco decline (mls/min/mmHg)	
			Number of ≥15% (proportion)	Number of ≥10% (proportion)	Mean	95% CI of difference between subgroups
Derivation cohort	Non-CPFE	132/183	52 (39.39%)	73 (55.30%)	645.39	-881.03~129.87*
	CPFE with emphysema <10%	125/174	42 (33.60%)	60 (48%)	1020.97	-752.33~301.34^#^
	CPFE with emphysema ≥10%	107/143	42 (39.25%)	59 (55.14%)	870.88	-683.49~383.31^^^
Replication cohort	Non-CPFE	108/152	30 (27.78%)	43 (39.81%)	769.10	-228.07~536.20*
	CPFE with emphysema <10%	161/206	38 (23.60%)	67 (41.61%)	615.04	-222.08~597.87^#^
	CPFE with emphysema ≥10%	117/152	42 (35.90%)	64 (54.70%)	581.21	-407.07~339.41^^^
Combined drug trial cohort	Non-CPFE	213/236	71 (33.33%)	100 (46.95%)	748.91	-450.51~220.82*
	CPFE with emphysema <10%	238/261	66 (27.73%)	112 (47.06%)	863.75	-448.18~316.55^#^
	CPFE with emphysema ≥10%	146/157	54 (36.99%)	80 (54.79%)	814.72	-423.13~325.08^^^

The proportions of patients with more than 15% and 10% relative 1-year DLco decline, and the mean of absolute 1-year DLco decline in derivation, replication cohorts and combined drug trial cohort (patients fulfilling criteria to enter IPF therapeutic trials in derivation and replication cohorts) are shown in this table. The number of subjects with available DLco decline versus the number of all subjects belonging to a certain subgroup is shown in n/n format. We also compared a) non-CPFE with CPFE with emphysema <10%, b) non-CPFE with CPFE with emphysema ≥10%, c) CPFE with emphysema ≥10% and CPFE with emphysema <10%, in terms of the relative decline and absolute decline. We use *, # and ^ to denote comparison a), b), c) respectively in the table. **None of the comparisons showed statistically significant differences**. CPFE: combined pulmonary fibrosis and emphysema; IPF: idiopathic pulmonary fibrosis; DLco: diffusing capacity of the lung for carbon monoxide; CI: confidence interval.

**Table 4:** Multivariable mixed-effects Cox proportional hazards regression models in non-CPFE patients and the two CPFE subgroups in the derivation IPF cohort.

Subgroup	Baseline severity and PFTs changes models	C-index	p-value	Hazard ratio	95% CI	
					Lower	Upper
Non-CPFE IPF patients (n=130, 61 deaths)	1-year FVC relative decline	0.821	3.02×10^-8^	1.082	1.052	1.113
	Binary 1-year FVC decline (5%)	0.805	1.09×10^-5^	3.824	2.104	6.953
	Binary 1-year FVC decline (10%)	0.811	4.96×10^-7^	4.261	2.422	7.497
	1-year DLco relative decline	0.803	0.0001	1.038	1.018	1.058
	Binary 1-year DLco decline (10%)	0.800	0.0010	2.764	1.511	5.055
	Binary 1-year DLco decline (15%)	0.811	4.69×10^-7^	4.211	2.407	7.366
CPFE patients with emphysema < 10% (n=119, 63 deaths)	1-year FVC relative decline	0.716	6.46×10^-5^	1.051	1.026	1.077
	Binary 1-year FVC decline (5%)	0.721	0.0001	3.000	1.705	5.279
	Binary 1-year FVC decline (10%)	0.685	0.025	1.983	1.091	3.604
	1 -year DLco relative decline	0.727	0.0003	1.035	1.016	1.055
	Binary 1-year DLco decline (10%)	0.682	0.173	1.453	0.849	2.486
	Binary 1-year DLco decline (15%)	0.696	0.017	1.979	1.131	3.464
CPFE patients with emphysema ≥1% (n=103, 73 deaths)	1-year FVC relative decline	0.714	0.008	1.034	1.009	1.061
	Binary 1-year FVC decline (5%)	0.714	0.016	1.868	1.126	3.100
	Binary 1-year FVC decline (10%)	0.715	0.002	2.540	1.421	4.539
	1-year DLco relative decline	0.732	1.24×10^-5^	1.033	1.018	1.049
	Binary 1-year DLco decline (1%)	0.703	0.058	1.619	0.983	2.665
	Binary 1-year DLco decline (15%)	0.732	7.61×10^-5^	2.674	1.643	4.353

Multivariable mixed-effects Cox regression models were used to investigate associations with mortality for 1-year FVC decline and 1-year DLco decline after adjusting for patient age, sex, smoking status (never versus ever), antifibrotic use (never versus ever) and baseline disease severity estimated using DLco. Binary 1-year FVC decline uses 5% and 10% relative decline as thresholds, and binary 1-year DLco decline uses 10% and 15% relative decline as thresholds. Separate centres/countries within the derivation cohort were modelled as multilevel with random effects between centres/countries (a random intercept per centre/country). All models passed Schoenfeld residuals test. CPFE: combined pulmonary fibrosis and emphysema; IPF: idiopathic pulmonary fibrosis; PFT: pulmonary function test; FVC: forced vital capacity; DLco: diffusing capacity of the lung for carbon monoxide; C-index: concordance index; CI: confidence interval.

**Table 5:** Multivariable mixed-effects Cox proportional hazards regression models in non-CPFE patients and the two CPFE subgroups in the replication IPF cohort.

Subgroup	Baseline severity and PFTs changes models	C-index	p-value	Hazard ratio	95% CI	
					Lower	Upper
Non-CPFE IPF patients (n=108, 45 deaths)	1-year FVC relative decline	0.823	8.65×10^-5^	1.086	1.042	1.132
	Binary 1-year FVC decline (5%)	0.827	0.002	2.719	1.425	5.187
	Binary 1-year FVC decline (10%)	0.817	0.004	2.733	1.374	5.437
	1 -year DLco relative decline	0.822	0.019	1.032	1.005	1.059
	Binary 1-year DLco decline (10%)	0.835	0.013	2.373	1.201	4.688
	Binary 1-year DLco decline (15%)	0.835	0.006	2.693	1.336	5.428
CPFE patients with emphysema < 10% (n=159, 83 deaths)	1-year FVC relative decline	0.754	0.001	1.055	1.022	1.089
	Binary 1-year FVC decline (5%)	0.763	0.004	1.960	1.246	3.083
	Binary 1-year FVC decline (10%)	0.767	9.27×10^-5^	2.704	1.642	4.453
	1 -year DLco relative decline	0.776	2.87×10^-5^	1.032	1.017	1.047
	Binary 1-year DLco decline (10%)	0.772	0.0005	2.252	1.424	3.561
	Binary 1-year DLco decline (15%)	0.768	0.0001	2.781	1.659	4.661
CPFE patients with emphysema ≥1% (n=115, 70 deaths)	1-year FVC relative decline	0.705	0.130	1.024	0.993	1.056
	Binary 1-year FVC decline (5%)	0.689	0.707	1.105	0.656	1.863
	Binary 1-year FVC decline (10%)	0.706	0.035	2.028	1.053	3.906
	1 -year DLco relative decline	0.720	0.001	1.030	1.012	1.049
	Binary 1-year DLco decline (10%)	0.716	0.0004	2.672	1.546	4.617
	Binary 1-year DLco decline (15%)	0.729	1.04×10^-5^	3.883	2.124	7.097

Multivariable mixed-effects Cox regression models were used to investigate associations with mortality for 1-year FVC decline and 1-year DLco decline after adjusting for patient age, sex, smoking status (never versus ever), antifibrotic use (never versus ever) and baseline disease severity estimated using DLco. Binary 1-year FVC decline uses 5% and 10% relative decline as thresholds, and binary 1-year DLco decline uses 10% and 15% relative decline as thresholds. Separate centres/countries within the replication cohort were modelled as multilevel with random effects between centres/countries (a random intercept per centre/country). All models passed Schoenfeld residuals test. CPFE: combined pulmonary fibrosis and emphysema; IPF: idiopathic pulmonary fibrosis; PFT: pulmonary function test; FVC: forced vital capacity; DLco: diffusing capacity of the lung for carbon monoxide; C-index: concordance index; CI: confidence interval.
