# Supplementary material for: Mortality surrogates in combined pulmonary fibrosis and emphysema
Source: Eur Respir J. Author manuscript; Available in PMC 2024 Jun 14. (PMC7616106; doi:10.1183/13993003.00127-2023)
Supplement: Supplementary Materials [file EMS196079-supplement-Supplementary_Materials.pdf]

Supplementary Appendix

Supplementary Table 1. Baseline characteristics of patients with and without longitudinal PFTs in derivation and replication cohorts.

Cohort	Variable	Subjects with longitudinal PFTs available	Subjects without longitudinal PFTs	p-value
Derivation cohort	Subjects (%)	356 (71.2)	144 (28.8)	-
	Age (years)	66.6±9.4	67.0±8.6	0.63
	Male (%)	279/356 (78.4)	106/144 (73.6)	0.30
	Never-/ever-smokers (ever %)	95/257 (73.0) *	43/101 (70.1)	0.59
	Visual fibrosis extent (%)	37.6±14.2	40.7±14.1	0.024
	Visual emphysema extent (%)	7.6±9.9	7.3±9.5	< 0.0001
	FVC (% predicted, n)	80.8±20.4 (356)	68.8±20.6 (74)	< 0.0001
	DLco (% predicted, n)	48.9±15.9 (356)	47.0±16.6 (49)	0.47
Replication cohort	Subjects (%)	385(75.5)	125(24.5)	-
	Age (years)	71.3±8.0	71.7±9.0	0.69
	Male (%)	303/385 (78.7)	89/125 (71.2)	0.11
	Never-/ever-smokers (ever %)	111/271 (70.9) †	40/84 (67.7) ††	0.57
	Visual fibrosis extent (%)	35.3±13.2	35.5±14.1	0.89
	Visual emphysema extent (%)	8.7±11.1	7.1±9.0	0.11
	FVC (% predicted, n)	85.0±19.2 (385)	85.5±24.8 (73)	0.87
	DLco (% predicted, n)	48.9±15.3 (385)	52.0±18.2 (38)	0.31

FVC: forced vital capacity; DLco: diffusing capacity of the lung for carbon monoxide; * 4 patients in the derivation cohort; † 3 patients and †† 1 patient in the replication cohort groups had no smoking data available. The p-value shows the significance of the difference between patients with and without longitudinal PFTs.

Supplementary Table 2. Baseline characteristics of non-CPFE IPF patients and CPFE patients fulfilling criteria to enter IPF therapeutic trials and with emphysema below or above 10% in the combined cohorts.

Variable	Non-CPFE IPF patients	CPFE patients with emphysema <10%	CPFE patients with emphysema ≥10%
Subjects (%)	236 (36.1)	261 (39.9)	157 (24.0)
Age (years)	69.8±8.2	69.8±8.8	67.5±9.0
Male (%)	141/236 (59.7)	209/261 (80.1)	142/157 (90.4)
Never-/ever-smokers (ever %)	121/115 (48.7)	64/192 (75) *	15/140 (90.3) **
Visual fibrosis extent (%)	34.4±13.9	33.9±12.9	37.5±13.0
Visual emphysema extent (%)	0±0	4.7±2.3	18.7±8.4
FVC (% predicted)	83.9±19.1	85.2±17.7	85.7±17.9
DLco (% predicted)	55.7±14.1	53.1±13.7	45.7±9.8

FVC: forced vital capacity; DLco: diffusing capacity of the lung for carbon monoxide; CPFE: combined pulmonary fibrosis and emphysema; IPF: idiopathic pulmonary fibrosis; * 256 patients and ** 155 patients had smoking data available.

Supplementary Table 3. Baseline characteristics of non-CPFE IPF patients and CPFE patients with emphysema below or above 15% in the derivation and replication cohorts.

Cohort	Variable	Non-CPFE IPF patients	CPFE patients with emphysema <15%	CPFE patients with emphysema ≥15%
Derivation cohort	Subjects (%)	183 (36.6)	218 (43.6)	99 (19.8)
	Age (years)	67.8±9.2	66.3±9.1	65.4±9.2
	Male (%)	110/183 (60.1)	185/218 (84.9)	90/99 (90.9)
	Never-/ever-smokers (ever %)	92/91 (49.7)	40/174 (81.3) *	6/93 (93.9)
	Visual fibrosis extent (%)	38.7±14.6	37.3±13.9	40.8±14.0
	Visual emphysema extent (%)	0±0	6.2±3.6	24.2±8.2
	FVC (% predicted, n)	77.1±20.8 (158)	78.7±20.4 (189)	81.7±22.1 (83)
	DLco (% predicted, n)	52.2±16.5 (151)	50.1±14.7 (174)	38.7±13.9 (80)
Replication cohort	Subjects (%)	152 (29.8)	258 (50.59)	100 (19.6)
	Age (years)	71.6±8.4	71.7±8.1	70.3±8.6
	Male (%)	96/152 (63.2)	211/258 (81.8)	85/100 (85)
	Never-/ever-smokers (ever %)	78/74 (48.7)	60/195 (76.5) †	13/86 (86.9) ††
	Visual fibrosis extent (%)	34.0±14.9	35.2±12.9	37.7±11.9
	Visual emphysema extent (%)	0±0	6.3±3.6	26.0±10.9
	FVC (% predicted, n)	84.5±21.1 (137)	84.3±20.4 (227)	87.8±18.3 (94)
	DLco (% predicted, n)	55.2±15.1 (121)	49.7±15.5 (215)	39.6±11.4 (87)

FVC: forced vital capacity; DLco: diffusing capacity of the lung for carbon monoxide; CPFE: combined pulmonary fibrosis and emphysema; IPF: idiopathic pulmonary fibrosis; * 214 patients had smoking data available in the derivation cohort; † 255 patients and †† 99 patients had smoking data available in the replication cohort.

Supplementary Table 4. Baseline characteristics of non-CPFE IPF patients and CPFE patients fulfilling criteria to enter IPF therapeutic trials and with emphysema below or above 15% in the combined cohorts.

Variable	Non-CPFE IPF patients	CPFE patients with emphysema <15%	CPFE patients with emphysema ≥15%
Subjects (%)	236 (36.1)	318 (48.6)	100 (15.3)
Age (years)	69.8±8.2	69.4±8.9	67.4±8.9
Male (%)	141/236 (59.7)	260/318 (81.8)	91/100 (91)
Never-/ever-smokers (ever %)	121/115 (48.7)	71/241 (77.2) *	8/91 (91.9) **
Visual fibrosis extent (%)	34.4±13.9	35.0±13.1	36.1±12.8
Visual emphysema extent (%)	0±0	6.0±3.5	22.6±8.2
FVC (% predicted)	83.9±19.1	84.7±17.7	87.5±17.8
DLco (% predicted)	55.7±14.1	52.0±13.1	45.0±10.4

FVC: forced vital capacity; DLco: diffusing capacity of the lung for carbon monoxide; CPFE: combined pulmonary fibrosis and emphysema; IPF: idiopathic pulmonary fibrosis; * 312 patients and ** 99 patients had smoking data available.

Supplementary Table 5. Baseline characteristics of non-CPFE IPF patients and CPFE patients in the *Fibrosis-Dominant CPFE* and *Matched-CPFE* subtypes in the derivation and replication cohorts.

Cohort	Variable	Non-CPFE IPF patients	*Fibrosis-Dominant CPFE* subtype	*Matched-CPFE* subtype
Derivation cohort	Subjects (%)	183 (36.6)	191 (38.2)	126 (25.2)
	Age (years)	67.8±9.2	66.7±9.1	65.0±9.1
	Male (%)	110/183 (60.1)	159/191 (83.2)	116/126 (92.1)
	Never-/ever-smokers (ever %)	92/91 (49.7)	40/148 (78.7) *	6/119 (95.2) **
	Visual fibrosis extent (%)	38.7±14.6	38.6±14.2	38.1±13.7
	Visual emphysema extent (%)	0±0	5.6±3.4	21.3±9.1
	FVC (% predicted, n)	77.1±20.8 (158)	78.3±19.9 (167)	81.8±22.5 (105)
	DLco (% predicted, n)	52.2±16.5 (151)	50.2±15.4 (153)	40.9±13.4 (101)
Replication cohort	Subjects (%)	152 (29.8)	227 (44.5)	131 (25.7)
	Age (years)	71.6±8.4	71.8±8.3	70.5±8.1
	Male (%)	96/152 (63.2)	187/227 (82.4)	109/131 (83.2)
	Never-/ever-smokers (ever %)	78/74 (48.7)	56/168 (75) †	17/113 (86.9) ††
	Visual fibrosis extent (%)	34.0±14.9	37.2±12.6	33.8±12.6
	Visual emphysema extent (%)	0±0	5.8±3.6	22.1±11.7
	FVC (% predicted, n)	84.5±21.1 (137)	83.1±20.4 (200)	88.9±18.4 (121)
	DLco (% predicted, n)	55.2±15.1 (121)	49.8±16.1 (189)	41.8±11.7 (113)

FVC: forced vital capacity; DLco: diffusing capacity of the lung for carbon monoxide; CPFE: combined pulmonary fibrosis and emphysema; IPF: idiopathic pulmonary fibrosis; *188 patients and **125 patients had smoking data available in derivation cohort; †224 patients and ††130 patients had smoking data available in replication cohort.

Supplementary Table 6. Baseline characteristics of non-CPFE IPF patients and *Fibrosis-Dominant CPFE* and *Matched-CPFE* subtypes fulfilling criteria to enter IPF therapeutic trials in the combined cohorts.

Variable	Non-CPFE IPF patients	*Fibrosis-Dominant CPFE* subtype	*Matched-CPFE* subtype
Subjects (%)	236 (36.1)	281 (43.0)	137 (20.9)
Age (years)	69.8±8.2	69.6±8.9	67.5±8.8
Male (%)	141/236 (59.7)	230/281 (81.9)	121/137 (88.3)
Never-/ever-smokers (ever %)	121/115 (48.7)	66/210 (76.1) *	13/122 (90.4) **
Visual fibrosis extent (%)	34.4±13.9	36.5±13.1	32.6±12.6
Visual emphysema extent (%)	0±0	5.4±3.4	19.2±9.0
FVC (% predicted)	83.9±19.1	84.3±17.6	87.7±17.7
DLco (% predicted)	55.7±14.1	52.1±13.7	46.7±10.0
FVC: forced vital capacity; DLco: diffusing capacity of the lung for carbon monoxide; CPFE: combined pulmonary fibrosis and emphysema; IPF: idiopathic pulmonary fibrosis; * 276 patients and ** 135 patients had smoking data available.			

Supplementary Table 7. FVC decline analysis in different subgroups of IPF patients.

Cohort	Subgroup	FVC data available cases/all case	Relative 1-year FVC decline (%)		Absolute 1-year FVC decline (mls)
			Number of ≥10% (proportion)	Number of ≥5% (proportion)	Mean
Derivation cohort	Non-CPFE	150/183	51 (34%)	81 (54%)	163.50
	CPFE with emphysema <15%	174/218	51 (29.31%)	90 (51.72%)	165.21
	CPFE with emphysema ≥15%	77/99	15 (19.48%)	28 (36.36%)	90.31
	Fibrosis-Dominant CPFE	153/191	46 (30.07%)	77 (50.33%)	159.50
	Matched-CPFE	98/126	20 (20.41%)	41 (41.84%)	115.27
Replication cohort	Non-CPFE	124/152	24 (19.35%)	50 (40.32%)	110.65
	CPFE with emphysema <15%	211/258	43 (20.38%)	91 (43.13%)	127.95
	CPFE with emphysema ≥15%	89/100	15 (16.85%)	28 (31.46%)	78.10
	Fibrosis-Dominant CPFE	187/227	41 (21.93%)	83 (44.39%)	135.32
	Matched-CPFE	113/131	17 (15.04%)	36 (31.86%)	76.48
Combined drug trial cohort	Non-CPFE	222/236	59 (26.58%)	105 (47.30%)	142.94
	CPFE with emphysema <15%	295/318	71 (24.07%)	141 (47.80%)*	161.88
	CPFE with emphysema ≥15%	95/100	15 (15.79%)	28 (29.47%)[†]	90.84
	Fibrosis-Dominant CPFE	262/281	65 (24.81%)	124 (47.33%)	163.21
	Matched-CPFE	128/137	21 (16.41%)	45 (35.16%)	106.42

The proportions of patients with more than 10% and 5% relative 1-year FVC decline, and the mean of absolute 1-year FVC decline in different subgroups in derivation, replication cohorts and combined drug trial cohort (patients fulfilling criteria to enter IPF therapeutic trials in derivation and replication cohorts) are shown in this table. The number of subjects with available FVC decline versus the number of all subjects within a subgroup is shown in n/n format. We also compared a) non-CPFE with CPFE with emphysema ≥15%, b) CPFE with emphysema ≥15% and CPFE with emphysema <15%, c) non-CPFE with *Matched-CPFE* subtype, d) *Fibrosis-Dominant CPFE* subtype and *Matched-CPFE* subtype in terms of the relative decline and absolute decline. CPFE: combined pulmonary fibrosis and emphysema; IPF: idiopathic pulmonary fibrosis; FVC: forced vital capacity; *=p<0.01 when comparing c); [†]=p<0.01 when comparing d).

Supplementary Table 8. DLco decline analysis in different subgroups of IPF patients.

Cohort	Subgroup	DLco data available cases/all case	Relative 1-year DLco decline (%)		Absolute 1-year DLco decline (mls/min/mmHg)
			Number of ≥15% (proportion)	Number of ≥10% (proportion)	Mean
Derivation cohort	Non-CPFE	132/183	52 (39.39%)	73 (55.30%)	645.39
	CPFE with emphysema <15%	157/218	51 (32.48%)	75 (47.77%)	950.61
	CPFE with emphysema ≥15%	75/99	33 (44.00%)	44 (58.67%)	954.13
	Fibrosis-Dominant CPFE	140/191	48 (34.29%)	67 (47.86%)	957.04
	Matched-CPFE	92/126	36 (39.13%)	52 (56.52%)	943.68
Replication cohort	Non-CPFE	108/152	30 (27.78%)	43 (39.81%)	769.10
	CPFE with emphysema <15%	197/258	51 (25.89%)	86 (43.65%)	617.02
	CPFE with emphysema ≥15%	81/100	29 (35.80%)	45 (55.56%)	561.34
	Fibrosis-Dominant CPFE	175/227	48 (27.43%)	81 (46.29%)	623.83
	Matched-CPFE	103/131	32 (31.07%)	50 (48.54%)	561.68
Combined drug trial cohort	Non-CPFE	213/236	71 (33.33%)	100 (46.95%)	748.91
	CPFE with emphysema <15%	291/318	83 (28.52%)	139 (47.77%)	832.87
	CPFE with emphysema ≥15%	93/100	37 (39.78%)	53 (56.99%)	883.39
	Fibrosis-Dominant CPFE	260/281	79 (30.38%)	128 (49.23%)	844.65
	Matched-CPFE	124/137	41 (33.06%)	64 (51.61%)	846.06

The proportions of patients with more than 15% and 10% relative 1-year DLco decline, and the mean of absolute 1-year DLco decline in different subgroups in derivation and replication cohorts and the combined drug trial cohort (patients fulfilling criteria to enter IPF therapeutic trials in derivation and replication cohorts) are shown in this table. The number of subjects with available DLco decline versus the number of all subjects within a subgroup is shown in n/n format. We also compared a) non-CPFE with CPFE with emphysema ≥15%, b) CPFE with emphysema ≥15% and CPFE with emphysema <15%, c) non-CPFE with *Matched-CPFE* subtype, d) *Fibrosis-Dominant CPFE* subtype and *Matched-CPFE* subtype in terms of the relative decline and absolute decline. **No statistically significant between group differences were identified.** CPFE: combined pulmonary fibrosis and emphysema; IPF: idiopathic pulmonary fibrosis; DLco: diffusing capacity of the lung for carbon monoxide.

Supplementary Table 9. Multivariable mixed-effects Cox proportional hazards regression models in non-CPFE patients and patients of the two CPFE subgroups (10% emphysema threshold) who fulfill criteria to enter IPF therapeutic trials in combined derivation and replication IPF cohorts.

Subgroup	Baseline severity and PFTs changes models	C-index	p-value	Hazard ratio	95% CI	
					Lower	Upper
Non-CPFE IPF patients (n=212, 87 deaths)	1-year FVC relative decline	0.812	1.29×10^{-11}	1.088	1.062	1.115
	Binary 1-year FVC decline (5%)	0.805	9.94×10^{-7}	3.268	2.034	5.252
	Binary 1-year FVC decline (10%)	0.807	2.13×10^{-9}	4.360	2.693	7.060
	1-year DLco relative decline	0.800	4.25×10^{-6}	1.042	1.024	1.06
	Binary 1-year DLco decline (10%)	0.805	6.23×10^{-5}	2.697	1.659	4.384
	Binary 1-year DLco decline (15%)	0.808	5.74×10^{-7}	3.337	2.081	5.352
CPFE patients with emphysema < 10% (n=233, 114 deaths)	1-year FVC relative decline	0.711	6.70×10^{-7}	1.049	1.03	1.069
	Binary 1-year FVC decline (5%)	0.710	0.0003	2.007	1.376	2.928
	Binary 1-year FVC decline (10%)	0.699	0.0001	2.282	1.502	3.469
	1-year DLco relative decline	0.735	6.20×10^{-9}	1.04	1.027	1.054
	Binary 1-year DLco decline (10%)	0.710	0.0002	2.110	1.429	3.116
	Binary 1-year DLco decline (15%)	0.719	5.87×10^{-7}	2.885	1.904	4.372
CPFE patients with emphysema ≥ 10% (n=144, 89 deaths)	1-year FVC relative decline	0.710	0.0006	1.051	1.022	1.082
	Binary 1-year FVC decline (5%)	0.700	0.022	1.693	1.077	2.660
	Binary 1-year FVC decline (10%)	0.708	0.001	2.363	1.412	3.955
	1-year DLco relative decline	0.723	5.45×10^{-8}	1.041	1.026	1.056
	Binary 1-year DLco decline (10%)	0.691	0.003	1.987	1.272	3.105
	Binary 1-year DLco decline (15%)	0.730	2.33×10^{-7}	3.376	2.129	5.353

Multivariable mixed-effects Cox regression models were used to investigate associations with mortality for 1-year FVC decline and 1-year DLco decline after adjusting for patient age, sex, smoking status (never versus ever), antifibrotic use (never versus ever) and baseline disease severity estimated using DLco. Binary 1-year FVC decline uses 5% and 10% relative decline as thresholds, and binary 1-year DLco decline uses 10% and 15% relative decline as thresholds. Separate centres/countries within the derivation and replication cohorts were modelled as multilevel with random effects between centres/countries (a random intercept per centre/country). All models passed Schoenfeld residuals test. CPFE: combined pulmonary fibrosis and emphysema; IPF: idiopathic pulmonary fibrosis; PFT: pulmonary function test; FVC: forced vital capacity; DLco: diffusing capacity of the lung for carbon monoxide; C-index: concordance index; CI: confidence interval.

Supplementary Table 10. Multivariable mixed-effects Cox proportional hazards regression models in non-CPFE patients and the two CPFE subgroups (15% emphysema threshold) in the derivation IPF cohort.

Subgroup	Baseline severity and PFTs changes models	C-index	p-value	Hazard ratio	95% CI	
					Lower	Upper
Non-CPFE IPF patients (n=130, 61 deaths)	1-year FVC relative decline	0.821	3.02×10^{-8}	1.082	1.052	1.113
	Binary 1-year FVC decline (5%)	0.805	1.09×10^{-5}	3.824	2.104	6.953
	Binary 1-year FVC decline (10%)	0.811	4.96×10^{-7}	4.261	2.422	7.497
	1-year DLco relative decline	0.803	0.0001	1.038	1.018	1.058
	Binary 1-year DLco decline (10%)	0.800	0.001	2.764	1.511	5.055
	Binary 1-year DLco decline (15%)	0.811	4.69×10^{-7}	4.211	2.407	7.366
CPFE patients with emphysema < 15% (n=149, 87 deaths)	1-year FVC relative decline	0.719	0.0003	1.037	1.016	1.057
	Binary 1-year FVC decline (5%)	0.722	0.0002	2.487	1.546	4.001
	Binary 1-year FVC decline (10%)	0.707	0.016	1.847	1.122	3.039
	1-year DLco relative decline	0.742	7.87×10^{-6}	1.038	1.021	1.055
	Binary 1-year DLco decline (10%)	0.707	0.075	1.510	0.960	2.377
	Binary 1-year DLco decline (15%)	0.725	0.0009	2.213	1.380	3.548
CPFE patients with emphysema ≥ 15% (n=73, 49 deaths)	1-year FVC relative decline	0.729	0.002	1.055	1.020	1.090
	Binary 1-year FVC decline (5%)	0.723	0.020	2.169	1.128	4.170
	Binary 1-year FVC decline (10%)	0.730	0.001	4.305	1.756	10.551
	1-year DLco relative decline	0.742	7.28×10^{-5}	1.034	1.017	1.051
	Binary 1-year DLco decline (10%)	0.720	0.057	1.842	0.983	3.451
	Binary 1-year DLco decline (15%)	0.738	0.0005	2.931	1.598	5.375

Multivariable mixed-effects Cox regression models were used to investigate associations with mortality for 1-year FVC decline and 1-year DLco decline after adjusting for patient age, sex, smoking status (never versus ever), antifibrotic use (never versus ever) and baseline disease severity estimated using DLco. Binary 1-year FVC decline uses 5% and 10% relative decline as thresholds, and binary 1-year DLco decline uses 10% and 15% relative decline as thresholds. Separate centres/countries within the derivation cohort were modelled as multilevel with random effects between centres/countries (a random intercept per centre/country). All models passed Schoenfeld residuals test. CPFE: combined pulmonary fibrosis and emphysema; IPF: idiopathic pulmonary fibrosis; PFT: pulmonary function test; FVC: forced vital capacity; DLco: diffusing capacity of the lung for carbon monoxide; C-index: concordance index; CI: confidence interval.

Supplementary Table 11. Multivariable mixed-effects Cox proportional hazards regression models in non-CPFE patients and the two CPFE subgroups (15% emphysema threshold) in the replication IPF cohort.

Subgroup	Baseline severity and PFTs changes models	C-index	p-value	Hazard ratio	95% CI	
					Lower	Upper
Non-CPFE IPF patients (n=108, 45 deaths)	1-year FVC relative decline	0.823	8.65×10^{-5}	1.086	1.042	1.132
	Binary 1-year FVC decline (5%)	0.827	0.002	2.719	1.425	5.187
	Binary 1-year FVC decline (10%)	0.817	0.004	2.733	1.374	5.437
	1-year DLco relative decline	0.822	0.019	1.032	1.005	1.059
	Binary 1-year DLco decline (10%)	0.835	0.013	2.373	1.201	4.688
	Binary 1-year DLco decline (15%)	0.835	0.006	2.693	1.336	5.428
CPFE patients with emphysema < 15% (n=194, 102 deaths)	1-year FVC relative decline	0.750	0.0005	1.053	1.023	1.085
	Binary 1-year FVC decline (5%)	0.754	0.002	1.890	1.260	2.835
	Binary 1-year FVC decline (10%)	0.760	2.44×10^{-5}	2.657	1.688	4.183
	1-year DLco relative decline	0.776	4.21×10^{-6}	1.032	1.018	1.047
	Binary 1-year DLco decline (10%)	0.766	0.0002	2.181	1.454	3.272
	Binary 1-year DLco decline (15%)	0.767	7.76×10^{-6}	2.798	1.782	4.393
CPFE patients with emphysema ≥ 15% (n=80, 51 deaths)	1-year FVC relative decline	0.722	0.122	1.027	0.993	1.063
	Binary 1-year FVC decline (5%)	0.688	0.865	1.056	0.565	1.973
	Binary 1-year FVC decline (10%)	0.706	0.079	2.052	0.920	4.576
	1-year DLco relative decline	0.720	0.010	1.026	1.006	1.047
	Binary 1-year DLco decline (10%)	0.709	0.0025	2.767	1.430	5.353
	Binary 1-year DLco decline (15%)	0.724	0.0003	3.846	1.866	7.925

Multivariable mixed-effects Cox regression models were used to investigate associations with mortality for 1-year FVC decline and 1-year DLco decline after adjusting for patient age, sex, smoking status (never versus ever), antifibrotic use (never versus ever) and baseline disease severity estimated using DLco. Binary 1-year FVC decline uses 5% and 10% relative decline as thresholds, and binary 1-year DLco decline uses 10% and 15% relative decline as thresholds. Separate centres/countries within the replication cohort were modelled as multilevel with random effects between centres/countries (a random intercept per centre/country). All models passed Schoenfeld residuals test. CPFE: combined pulmonary fibrosis and emphysema; IPF: idiopathic pulmonary fibrosis; PFT: pulmonary function test; FVC: forced vital capacity; DLco: diffusing capacity of the lung for carbon monoxide; C-index: concordance index; CI: confidence interval.

Supplementary Table 12. Multivariable mixed-effects Cox proportional hazards regression models in non-CPFE patients and patients of the two CPFE subgroups (15% emphysema threshold) who fulfill criteria to enter IPF therapeutic trials in combined derivation and replication IPF cohorts.

Subgroup	Baseline severity and PFTs changes models	C-index	p-value	Hazard ratio	95% CI	
					Lower	Upper
Non-CPFE IPF patients (n=212, 87 deaths)	1-year FVC relative decline	0.812	1.29×10^{-11}	1.088	1.062	1.115
	Binary 1-year FVC decline (5%)	0.805	9.94×10^{-7}	3.268	2.034	5.252
	Binary 1-year FVC decline (10%)	0.807	2.13×10^{-9}	4.36	2.693	7.06
	1-year DLco relative decline	0.800	4.25×10^{-6}	1.042	1.024	1.06
	Binary 1-year DLco decline (10%)	0.805	6.23×10^{-5}	2.697	1.659	4.384
	Binary 1-year DLco decline (15%)	0.808	5.74×10^{-7}	3.337	2.081	5.352
CPFE patients with emphysema < 15% (n=285, 147 deaths)	1-year FVC relative decline	0.721	4.51×10^{-7}	1.045	1.028	1.064
	Binary 1-year FVC decline (5%)	0.720	0.0001	1.913	1.370	2.671
	Binary 1-year FVC decline (10%)	0.714	6.63×10^{-6}	2.356	1.623	3.42
	1-year DLco relative decline	0.760	5.28×10^{-13}	1.046	1.034	1.059
	Binary 1-year DLco decline (10%)	0.730	1.50×10^{-5}	2.127	1.511	2.994
	Binary 1-year DLco decline (15%)	0.739	2.99×10^{-10}	3.199	2.228	4.593
CPFE patients with emphysema ≥ 15% (n=92, 56 deaths)	1-year FVC relative decline	0.735	0.0004	1.071	1.031	1.112
	Binary 1-year FVC decline (5%)	0.722	0.025	2.030	1.091	3.777
	Binary 1-year FVC decline (10%)	0.717	0.009	2.764	1.295	5.899
	1-year DLco relative decline	0.714	0.0009	1.029	1.012	1.047
	Binary 1-year DLco decline (10%)	0.689	0.077	1.701	0.945	3.061
	Binary 1-year DLco decline (15%)	0.720	0.001	2.623	1.478	4.657

Multivariable mixed-effects Cox regression models were used to investigate associations with mortality for 1-year FVC decline and 1-year DLco decline after adjusting for patient age, sex, smoking status (never versus ever), antifibrotic use (never versus ever) and baseline disease severity estimated using DLco. Binary 1-year FVC decline uses 5% and 10% relative decline as thresholds, and binary 1-year DLco decline uses 10% and 15% relative decline as thresholds. Separate centres/countries within the derivation and replication cohorts were modelled as multilevel with random effects between centres/countries (a random intercept per centre/country). All models passed Schoenfeld residuals test. CPFE: combined pulmonary fibrosis and emphysema; IPF: idiopathic pulmonary fibrosis; PFT: pulmonary function test; FVC: forced vital capacity; DLco: diffusing capacity of the lung for carbon monoxide; C-index: concordance index; CI: confidence interval.

Supplementary Table 13. Multivariable mixed-effects Cox proportional hazards regression models in non-CPFE patients and the two CPFE SuStaIn subtypes in the derivation IPF cohort.

Subgroup	Baseline severity and PFTs changes models	C-index	p-value	Hazard ratio	95% CI	
					Lower	Upper
Non-CPFE IPF patients (n=130, 61 deaths)	1-year FVC relative decline	0.821	3.02×10^{-8}	1.082	1.052	1.113
	Binary 1-year FVC decline (5%)	0.805	1.09×10^{-5}	3.824	2.104	6.953
	Binary 1-year FVC decline (10%)	0.811	4.96×10^{-7}	4.261	2.422	7.497
	1-year DLco relative decline	0.803	0.0001	1.038	1.018	1.058
	Binary 1-year DLco decline (10%)	0.800	0.001	2.764	1.511	5.055
	Binary 1-year DLco decline (15%)	0.811	4.69×10^{-7}	4.211	2.407	7.366
Fibrosis-Dominant CPFE patients (n=134, 76 deaths)	1-year FVC relative decline	0.731	0.0005	1.039	1.017	1.062
	Binary 1-year FVC decline (5%)	0.743	7.82×10^{-5}	2.765	1.669	4.580
	Binary 1-year FVC decline (10%)	0.718	0.009	2.018	1.189	3.424
	1-year DLco relative decline	0.745	0.0001	1.033	1.016	1.051
	Binary 1-year DLco decline (10%)	0.719	0.0831	1.540	0.945	2.509
	Binary 1-year DLco decline (15%)	0.732	0.003	2.168	1.313	3.577
Matched-CPFE patients (n=88, 60 deaths)	1-year FVC relative decline	0.701	0.0064	1.040	1.011	1.070
	Binary 1-year FVC decline (5%)	0.704	0.059	1.711	0.980	2.987
	Binary 1-year FVC decline (10%)	0.705	0.012	2.484	1.219	5.065
	1-year DLco relative decline	0.727	1.07×10^{-5}	1.036	1.020	1.053
	Binary 1-year DLco decline (10%)	0.688	0.070	1.674	0.959	2.922
	Binary 1-year DLco decline (15%)	0.721	0.0004	2.634	1.535	4.518

Multivariable mixed-effects Cox regression models were used to investigate associations with mortality for 1-year FVC decline and 1-year DLco decline after adjusting for patient age, sex, smoking status (never versus ever), antifibrotic use (never versus ever) and baseline disease severity estimated using DLco. Binary 1-year FVC decline uses 5% and 10% relative decline as thresholds, and binary 1-year DLco decline uses 10% and 15% relative decline as thresholds. Separate centres/countries within the derivation cohort were modelled as multilevel with random effects between centres/countries (a random intercept per centre/country). All models passed Schoenfeld residuals test. CPFE: combined pulmonary fibrosis and emphysema; IPF: idiopathic pulmonary fibrosis; PFT: pulmonary function test; FVC: forced vital capacity; DLco: diffusing capacity of the lung for carbon monoxide; C-index: concordance index; CI: confidence interval.

Supplementary Table 14. Multivariable mixed-effects Cox proportional hazards regression models in non-CPFE patients and the two CPFE SuStaIn subtypes in the replication IPF cohort.

Subgroup	Baseline severity and PFTs changes models	C-index	p-value	Hazard ratio	95% CI	
					Lower	Upper
Non-CPFE IPF patients (n=108, 45 deaths)	1-year FVC relative decline	0.823	8.65×10^{-5}	1.086	1.042	1.132
Non-CPFE IPF patients (n=108, 45 deaths)	Binary 1-year FVC decline (5%)	0.827	0.002	2.719	1.425	5.187
Non-CPFE IPF patients (n=108, 45 deaths)	Binary 1-year FVC decline (10%)	0.817	0.004	2.733	1.374	5.437
Non-CPFE IPF patients (n=108, 45 deaths)	1-year DLco relative decline	0.822	0.019	1.032	1.005	1.059
Non-CPFE IPF patients (n=108, 45 deaths)	Binary 1-year DLco decline (10%)	0.835	0.013	2.373	1.201	4.688
Non-CPFE IPF patients (n=108, 45 deaths)	Binary 1-year DLco decline (15%)	0.835	0.006	2.693	1.336	5.428
Fibrosis-Dominant CPFE patients (n=173, 95 deaths)	1-year FVC relative decline	0.764	0.0008	1.051	1.021	1.082
Fibrosis-Dominant CPFE patients (n=173, 95 deaths)	Binary 1-year FVC decline (5%)	0.765	0.0095	1.750	1.147	2.671
Fibrosis-Dominant CPFE patients (n=173, 95 deaths)	Binary 1-year FVC decline (10%)	0.770	0.0003	2.396	1.497	3.836
Fibrosis-Dominant CPFE patients (n=173, 95 deaths)	1-year DLco relative decline	0.782	9.06×10^{-5}	1.028	1.014	1.042
Fibrosis-Dominant CPFE patients (n=173, 95 deaths)	Binary 1-year DLco decline (10%)	0.772	1.82×10^{-10}	0.941	0.924	0.959
Fibrosis-Dominant CPFE patients (n=173, 95 deaths)	Binary 1-year DLco decline (15%)	0.772	0.0003	2.363	1.480	3.771
Matched-CPFE patients (n=101, 58 deaths)	1-year FVC relative decline	0.719	0.226	1.021	0.987	1.056
Matched-CPFE patients (n=101, 58 deaths)	Binary 1-year FVC decline (5%)	0.708	0.719	1.112	0.624	1.982
Matched-CPFE patients (n=101, 58 deaths)	Binary 1-year FVC decline (10%)	0.729	0.021	2.361	1.137	4.906
Matched-CPFE patients (n=101, 58 deaths)	1-year DLco relative decline	0.745	0.001	1.033	1.013	1.054
Matched-CPFE patients (n=101, 58 deaths)	Binary 1-year DLco decline (10%)	0.747	0.0001	3.468	1.845	6.517
Matched-CPFE patients (n=101, 58 deaths)	Binary 1-year DLco decline (15%)	0.764	1.33×10^{-5}	4.858	2.385	9.895

Multivariable mixed-effects Cox regression models were used to investigate associations with mortality for 1-year FVC decline and 1-year DLco decline after adjusting for patient age, sex, smoking status (never versus ever), antifibrotic use (never versus ever) and baseline disease severity estimated using DLco. Binary 1-year FVC decline uses 5% and 10% relative decline as thresholds, and binary 1-year DLco decline uses 10% and 15% relative decline as thresholds. Separate centres/countries within the replication cohort were modelled as multilevel with random effects between centres/countries (a random intercept per centre/country). All models passed Schoenfeld residuals test. CPFE: combined pulmonary fibrosis and emphysema; IPF: idiopathic pulmonary fibrosis; PFT: pulmonary function test; FVC: forced vital capacity; DLco: diffusing capacity of the lung for carbon monoxide; C-index: concordance index; CI: confidence interval.

Supplementary Table 15. Multivariable mixed-effects Cox proportional hazards regression models in non-CPFE patients and patients of the two CPFE SuStaIn subtypes who fulfill criteria to enter IPF therapeutic trials in combined derivation and replication IPF cohorts.

Subgroup	Baseline severity and PFTs changes models	C-index	p-value	Hazard ratio	95% CI	
					Lower	Upper
Non-CPFE IPF patients (n=212, 87 deaths)	1-year FVC relative decline	0.812	1.29×10^{-11}	1.088	1.062	1.115
	Binary 1-year FVC decline (5%)	0.805	9.94×10^{-7}	3.268	2.034	5.252
	Binary 1-year FVC decline (10%)	0.807	2.13×10^{-9}	4.36	2.693	7.06
	1-year DLco relative decline	0.800	4.25×10^{-6}	1.042	1.024	1.06
	Binary 1-year DLco decline (10%)	0.805	6.23×10^{-5}	2.697	1.659	4.384
	Binary 1-year DLco decline (15%)	0.808	5.74×10^{-7}	3.337	2.081	5.352
Fibrosis-Dominant CPFE patients (n=255, 131 deaths)	1-year FVC relative decline	0.727	5.19×10^{-6}	1.045	1.025	1.064
	Binary 1-year FVC decline (5%)	0.730	0.0005	1.877	1.319	2.671
	Binary 1-year FVC decline (10%)	0.721	6.12×10^{-5}	2.243	1.511	3.331
	1-year DLco relative decline	0.759	3.37×10^{-10}	1.042	1.028	1.055
	Binary 1-year DLco decline (10%)	0.734	0.0001	2.028	1.417	2.901
	Binary 1-year Dlco decline (15%)	0.741	9.46×10^{-9}	3.009	2.066	4.384
Matched-CPFE patients (n=122, 72 deaths)	1-year FVC relative decline	0.696	0.0006	1.058	1.025	1.093
	Binary 1-year FVC decline (5%)	0.680	0.051	1.663	0.998	2.772
	Binary 1-year FVC decline (10%)	0.686	0.002	2.669	1.420	5.015
	1-year DLco relative decline	0.722	1.39×10^{-7}	1.041	1.025	1.056
	Binary 1-year DLco decline (10%)	0.684	0.0007	2.412	1.453	4.006
	Binary 1-year DLco decline (15%)	0.730	9.58×10^{-7}	3.606	2.159	6.023

Multivariable mixed-effects Cox regression models were used to investigate associations with mortality for 1-year FVC decline and 1-year DLco decline after adjusting for patient age, sex, smoking status (never versus ever), antifibrotic use (never versus ever) and baseline disease severity estimated using DLco. Binary 1-year FVC decline uses 5% and 10% relative decline as thresholds, and binary 1-year DLco decline uses 10% and 15% relative decline as thresholds. Separate centres/countries within the derivation and replication cohorts were modelled as multilevel with random effects between centres/countries (a random intercept per centre/country). All models passed Schoenfeld residuals test. CPFE: combined pulmonary fibrosis and emphysema; IPF: idiopathic pulmonary fibrosis; PFT: pulmonary function test; FVC: forced vital capacity; DLco: diffusing capacity of the lung for carbon monoxide; C-index: concordance index; CI: confidence interval.

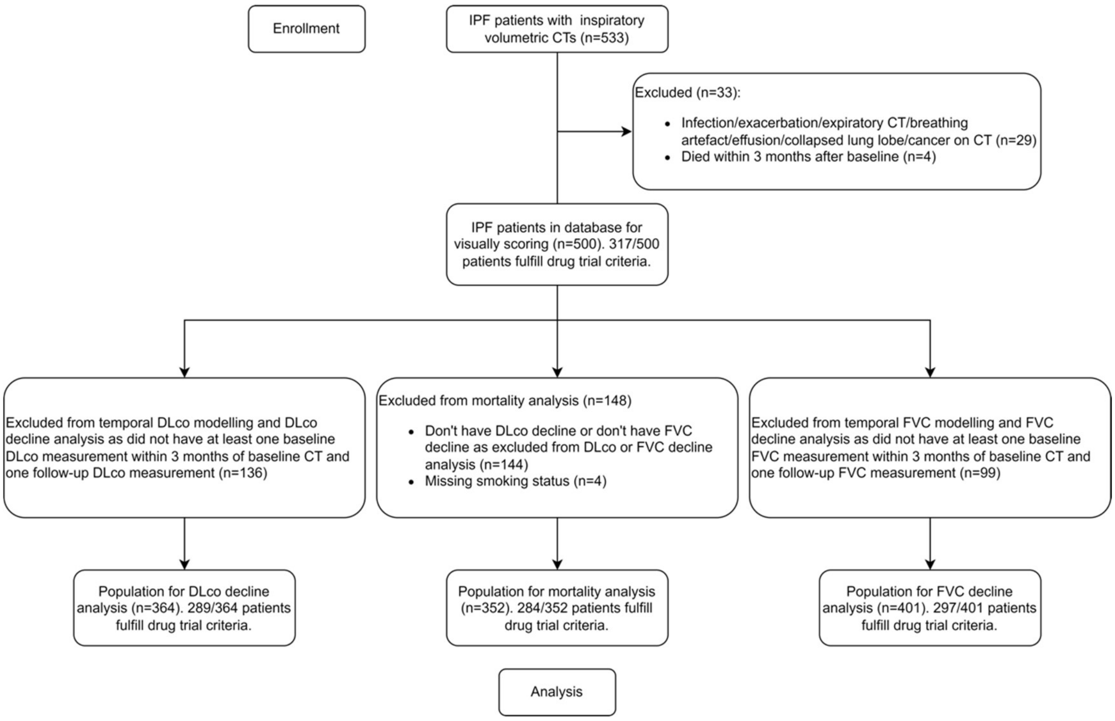

(a)

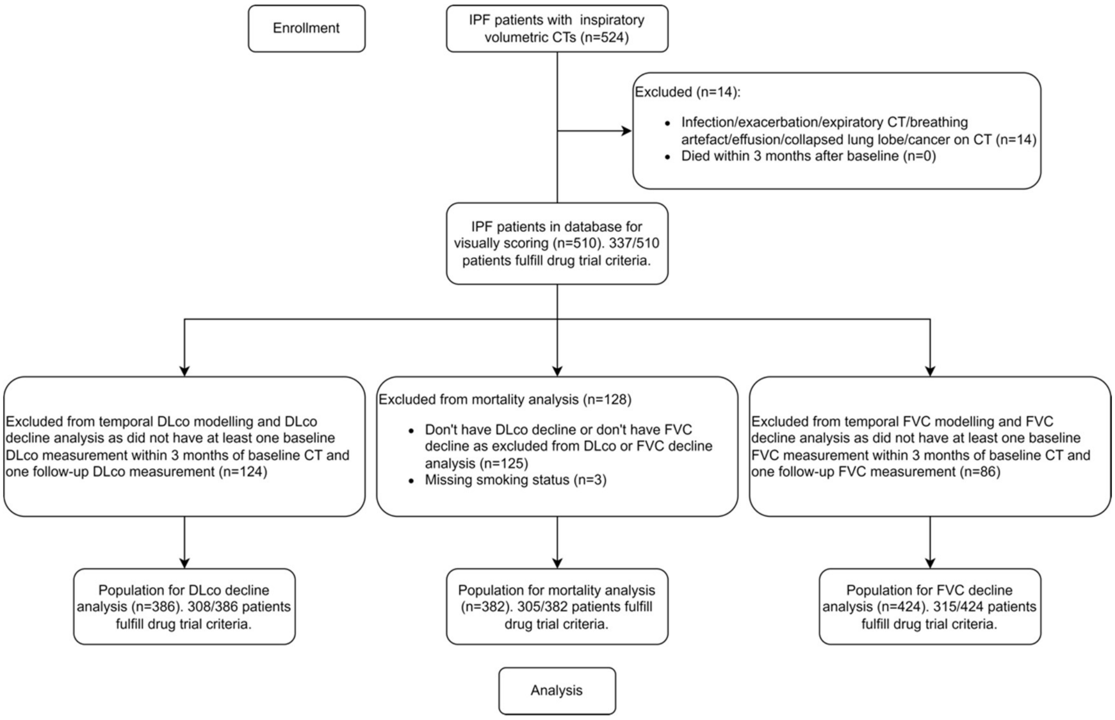

(b)

Supplementary Figure 1. CONSORT diagrams of derivation cohort (a) and replication cohort (b).

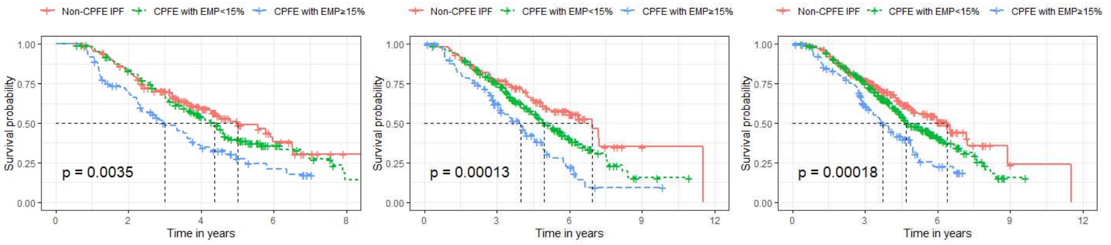

(a) (b) (c)

Supplementary Figure 2. Kaplan-Meier curves of non-CPFE IPF patients (red), CPFE patients with emphysema <15% (green) and CPFE patients with emphysema ≥ 15% (blue) in the derivation cohort (a), the replication cohort (b), combined derivation and replication cohort patients qualifying for therapeutic trials (c). Log-rank tests show a significant difference in mortality between the three subtypes in all three analyses.

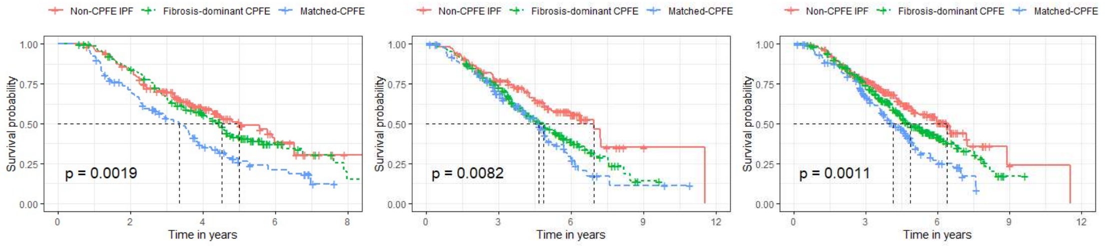

(a) (b) (c)

Supplementary Figure 3. Kaplan-Meier curves of non-CPFE IPF patients (red), *Fibrosis-Dominant CPFE* patients (green) and *Matched-CPFE* patients (blue) in the derivation cohort (a), the replication cohort (b), combined derivation and replication cohort patients qualifying for therapeutic trials (c). Log-rank tests show a significant difference in mortality between the three subtypes in all three analyses.
